# Autophagy compensates impaired energy metabolism in CLPXP‐deficient *Podospora anserina* strains and extends healthspan

**DOI:** 10.1111/acel.12600

**Published:** 2017-04-27

**Authors:** Laura Knuppertz, Heinz D. Osiewacz

**Affiliations:** ^1^ Institute of Molecular Biosciences and Cluster of Excellence ‘Macromolecular Complexes’ Department of Biosciences J. W. Goethe University Frankfurt Germany

**Keywords:** aging, autophagy, CLPXP protease, energy metabolism, mitochondria, *Podospora anserina*

## Abstract

The degradation of nonfunctional mitochondrial proteins is of fundamental relevance for maintenance of cellular homeostasis. The heteromeric CLPXP protein complex in the mitochondrial matrix is part of this process. In the fungal aging model *Podospora anserina*, ablation of CLPXP leads to an increase in healthy lifespan. Here, we report that this counterintuitive increase depends on a functional autophagy machinery. In *PaClpXP* mutants, autophagy is involved in energy conservation and the compensation of impairments in respiration. Strikingly, despite the impact on mitochondrial function, it is not mitophagy but general autophagy that is constitutively induced and required for longevity. In contrast, in another long‐lived mutant ablated for the mitochondrial PaIAP protease, autophagy is neither induced nor required for lifespan extension. Our data provide novel mechanistic insights into the capacity of different forms of autophagy to compensate impairments of specific components of the complex mitochondrial quality control network and about the biological role of mitochondrial CLPXP in the control of cellular energy metabolism.

## Introduction

Mitochondria are eukaryotic organelles involved in various essential functions including iron/sulfur cluster synthesis, lipid and amino acid metabolism, copper homeostasis, and energy transduction. Maintenance of mitochondrial homeostasis crucially depends on the control of protein quality by repair enzymes, chaperones, proteases, and autophagy. One mitochondrial matrix protease is ‘caseinolytic protease’ (CLP). This protein is active in prokaryotes as well as in mitochondria and plastids of most eukaryotes. All characterized CLP proteases have the same basic structure and function. They consist of two heptameric rings with individual units of the CLPP serine protease and form a proteolytic core cylinder, which is able to degrade small peptides and some unfolded proteins (Gottesman *et al*., 1997). Larger proteins which are degraded by CLPP are recognized, unfolded, and delivered to the proteolytic chamber of CLPP by CLPX, a hexameric AAA+ chaperone that together with two CLPP rings constitutes the CLPXP multiprotein complex. Until today, the precise biological role of this complex is only initially elucidated.

In the nematode *Caenorhabditis elegans*, CLPP was demonstrated to signal mitochondrial perturbation to the nucleus (Haynes *et al*., 2007) and induce the so‐called mitochondrial unfolded protein response (UPR^mt^). In mammals, the role of CLPP in the control of UPR^mt^ is less clear and controversially discussed. For instance, it was found that *ClpX* is selectively upregulated during myogenesis in mice, demonstrating the initiation of a CLPXP‐dependent UPR^mt^‐like response (Al‐Furoukh *et al*., 2015). In contrast, in a mouse model lacking a mitochondrial tRNA synthetase, it was shown that the UPR^mt^ is independent of CLPP (Seiferling *et al*., 2016).

In humans, a change in CLPXP abundance is associated with the development of different diseases. Perrault syndrome is one of these diseases, which is characterized by ovarian failure and sensorineural deafness (Jenkinson *et al*., 2013). *ClpP* null mice are a good model for this disease. They display prominent phenotypes including complete infertility and auditory deficits, growth retardation, induction of inflammatory factors, and resistance to ulcerative dermatitis (Gispert *et al*., 2013). Another example is Friedreich ataxia (FRDA), a neurodegenerative disease caused by failure to assemble Fe‐S clusters due to defects in the mitochondrial iron chaperone frataxin (Puccio & Koenig, 2002). In a cardiac conditional FRDA mouse model with a tissue‐targeted frataxin deficiency in striated muscles, the proteolytic component CLPP is upregulated at mid‐stage of the disease (Guillon *et al*., 2009). Finally, a role of CLPP in a number of cancers is suggested by an altered abundance of *ClpP* transcripts in different human cancer tissues, which may result from altered metabolic states in cancer cells (Goard & Schimmer, 2014; Cole *et al*., 2015; Seo *et al*., 2016).

In a previous study with the fungal aging model *Podospora anserina*, deletion of *PaClpP*, a gene encoding the proteolytic subunit of CLPXP, led to a counterintuitive pronounced increase in the healthy lifespan (‘healthspan’). In this mutant, lifespan increase is not linked to impairments in viability parameters (e.g., reduced growth rate and fertility) as it is typical for many long‐lived *P. anserina* mutants in which the composition of the mitochondrial respiratory chain is affected (Scheckhuber & Osiewacz, 2008). The phenotype of the *PaClpP* deletion mutant can be reverted by the human CLPP homolog (Fischer *et al*., 2013). A recent ‘CLPP substrate trapping assay’ resulted in the identification of 19 potential PaCLPP substrates and 47 interaction partners (Fischer *et al*., 2015). Most of them are proteins involved in the control of energy metabolism including components associated with components of metabolic pathways in mitochondria (e.g., pyruvate dehydrogenase complex, tricarboxylic acid cycle, subunits of electron transport chain complex I). These data suggest a function of mitochondrial CLPXP in the maintenance of energy metabolism. The same general role was concluded for mammalian CLPP (Cole *et al*., 2015; Seo *et al*., 2016; Szczepanowska *et al*., 2016). In addition, a moderate respiratory deficiency in CLPP knockout mice linked to ineffective mitochondrial protein synthesis caused by decreased amounts of fully assembled mitochondrial ribosomes (55S, mitoribosomes) was reported (Szczepanowska *et al*., 2016). Overall, the data support a basic functional conservation of CLPXP from fungi to mammals.

The observed healthspan extension in the *PaClpP* deletion strain raises the question about the molecular basis of this unexpected phenotype. Here, we demonstrate that the loss of functional PaCLPXP causes impairments in mitochondrial respiratory chain and induces compensatory responses. Moreover, we describe that lifespan extension in the *∆PaClpX* and *∆PaClpP* single mutants, and the *∆PaClpX/∆PaClpP* double mutant (hereafter termed ∆*PaClpXP*) depends on a functional autophagy machinery.

## Results

### Deletion of *PaClpXP* alters the composition of mitochondrial electron transport chain complexes and affects mitochondrial oxygen consumption

**Figure 1:**
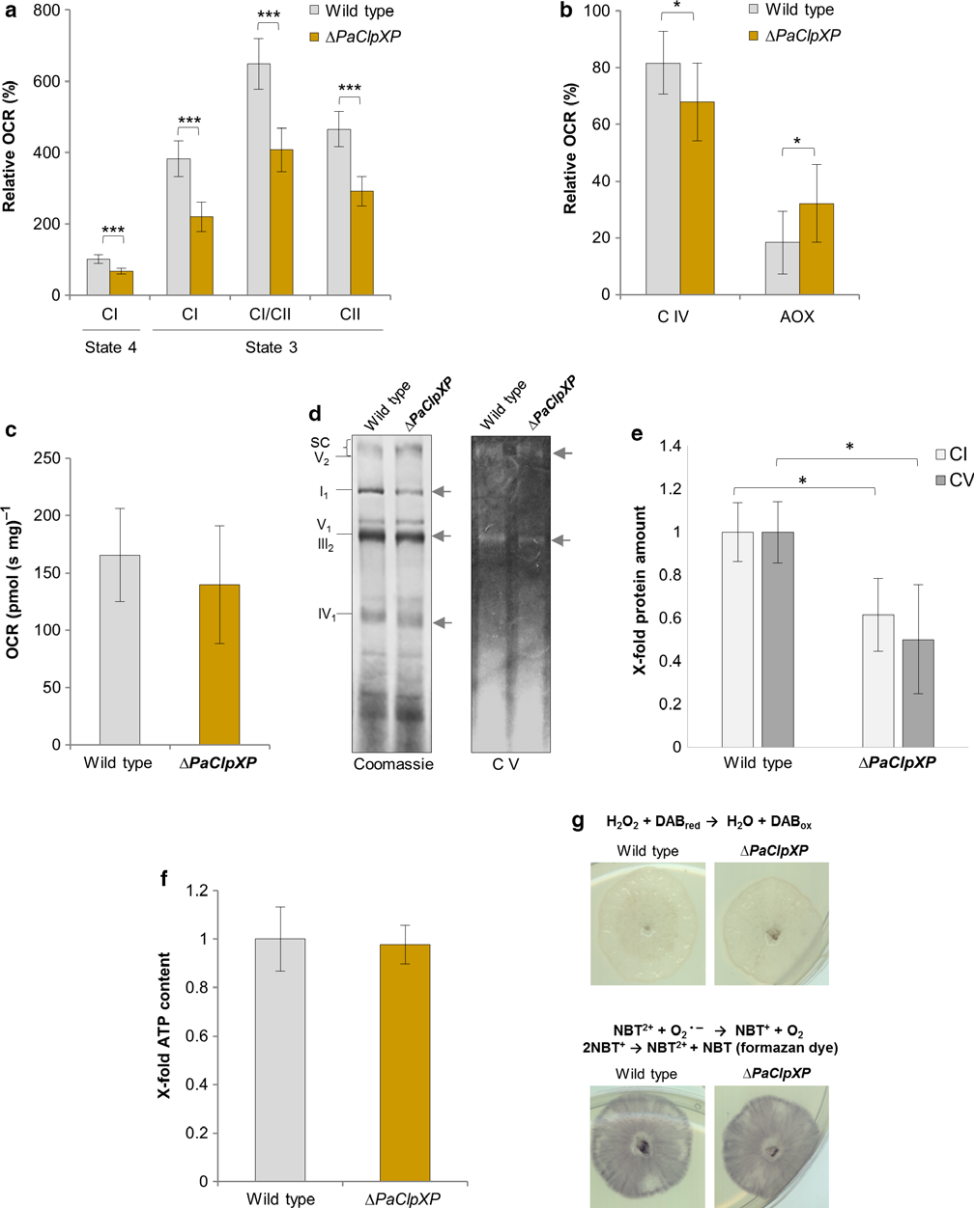
Mitochondrial function is partially impaired in *∆PaClpXP* compared to wild type. (a) Relative oxygen consumption rate (OCR) of 7‐day‐old *∆PaClpXP* and wild type mitochondria (for each strain, three mitochondrial preparations with three to six technical replicates were analyzed). State 4 OCR of the wild type was set to 100%. (b) The relative oxygen consumption of mycelia from 7‐day‐old wild type and *∆PaClpXP* strains (three biological and four technical replicates) was analyzed after KCN (inhibition of C IV) or SHAM [inhibition of alternative oxidase (AOX)] addition. The basal oxygen consumption of the wild type was set to 100%, and the inhibition of the oxidases was calculated relatively. (c) Basal oxygen consumption rate (OCR) per mg dry weight from mycelia of 7‐day‐old wild type and *∆PaClpXP* strains (three biological and four technical replicates). (d) BN‐PAGE analysis of 100 μg mitochondrial protein extracts from 7‐day‐old wild type and *∆PaClpXP* (three mitochondrial preparations). SC, supercomplexes, V_2_, complex V dimer; I_1_, complex I monomer; V_1_, complex V monomer; III_2_, complex III dimer; IV_1_, complex IV monomer. Changes in the composition are marked with arrows. Coomassie‐stained gel (left) and ‘in‐gel’ staining of C V (right) are demonstrated. (e) Quantification of relative complex I (CI, Coomassie‐stained gel) and complex V (CV, CV‐stained gel) protein levels of wild type and *∆PaClpXP* mitochondrial extracts (*n* = 3) that were separated during a BN‐PAGE analysis shown in (d). Protein abundance in the wild type was set to 1. Error bars correspond to the standard deviation. *P*‐values were determined by Student's *t*‐test (*P *<* *0.05). (f) ATP levels of 7‐day‐old wild type vs*. ∆PaClpXP* (three biological replicates with three technical replicates) were measured by a luminescence‐based assay. Error bars correspond to the standard deviation, and *P*‐values were determined with paired Student's *t‐*test (*P *>* *0.05). (g) Determination of superoxide and hydrogen peroxide in 7‐day‐old wild type and *∆PaClpXP* strains by NBT and DAB staining. (a–c) Error bars correspond to the standard deviation, and *P*‐values were determined by two‐tailed Mann–Whitney–Wilcoxon *U*‐test. (a, b, e) *= *P*< 0.05, **= *P*< 0.01, *** *P*< 0.001.

The recent identification of PaCLPP substrates and interaction partners suggested a role of PaCLPXP in the control of mitochondrial metabolic pathways (Fischer *et al*., 2015). To verify this function experimentally, we analyzed mitochondrial respiration in the wild type and the *∆PaClpXP* double mutant. In a first set of experiments, we determined the relative mitochondrial oxygen consumption rate (OCR) in isolated mitochondria. In this study, state 4 OCR of the wild type was set to 100%. Interestingly, when comparing both strains using pyruvate/malate as substrates of complex I (C I), mitochondrial oxygen consumption was reduced in the mutant in both state 4 and state 3 (Fig. 1a). Such a decrease in state 3 was also observed when succinate was additionally added as a substrate for complex II (C II; CI and CII respiration) and when complex I was inhibited by rotenone (C II respiration) (Fig. 1a). These results indicate a general and not complex‐specific decline of mitochondrial respiration in the *∆PaClpXP* strain.

Next, we analyzed mitochondrial respiration at the organismic level using mycelia of the wild type and *∆PaClpXP*. Normally, wild‐type strains of *P. anserina* respire via a standard cytochrome‐*c* oxidase (COX, complex IV)‐dependent respiratory chain. However, when this pathway is impaired for any reason, a compensating mechanism is induced leading to the expression of a gene coding for an alternative oxidase (AOX; Borghouts *et al*., 2001; Scheckhuber *et al*., 2011). To elucidate the contribution of these two terminal oxidases in the *∆PaClpXP* mutant, we measured the ratio between complex IV (C IV)‐ and AOX‐dependent OCR in mycelia of both strains. The basal OCR per mg dry weight of the wild type was set to 100%, and the relative inhibition of COX by cyanide or AOX by salicylhydroxamic acid (SHAM) was calculated (Fig. 1b). Interestingly, compared to the wild type, COX‐dependent respiration is reduced and respiration via AOX is increased by approximately 15% in *∆PaClpXP* (Fig. 1b). Basal OCR was not significantly changed compared to wild type (Fig. 1c). We verified these conclusions by western blot analysis which demonstrated that not only enzymatic activity but also the abundance of the AOX is increased in *∆PaClpXP* in comparison with wild type (Fig.S2).

To test whether the observed change in OCR results from the decreased abundance of individual respiratory complexes, blue native polyacrylamide gels (BN‐PAGE) with crude mitochondrial extracts of *∆PaClpXP* and the wild type (Fig. 1d) were analyzed. Concordantly, there is a decrease in the amount of C I and C V (Fig. 1d,e), as well as a slight mobility shift of C IV visualized by Coomassie staining. In addition, we analyzed the ‘in‐gel’ activity of C V. In *∆PaClpXP,* C V activity appears to be reduced (Fig. 1d,e). Unexpectedly, but in concordance with the healthy phenotype of the mutant, ATP content was unaltered in the *∆PaClpXP* strain (Fig. 1f), indicating that the observed changes in the mitochondrial respiratory chain are compensated. This conclusion is supported by the demonstration that neither superoxide levels (NBT staining), a ROS generated at the mitochondrial respiratory chain, nor hydrogen peroxide levels (DAB staining) generated by superoxide dismutation differ in *∆PaClpXP* compared to wild type (Fig. 1g).

### In *∆PaClpXP,* mitophagy is not induced as an adaptive response to impairments in protein quality control

**Figure 2:**
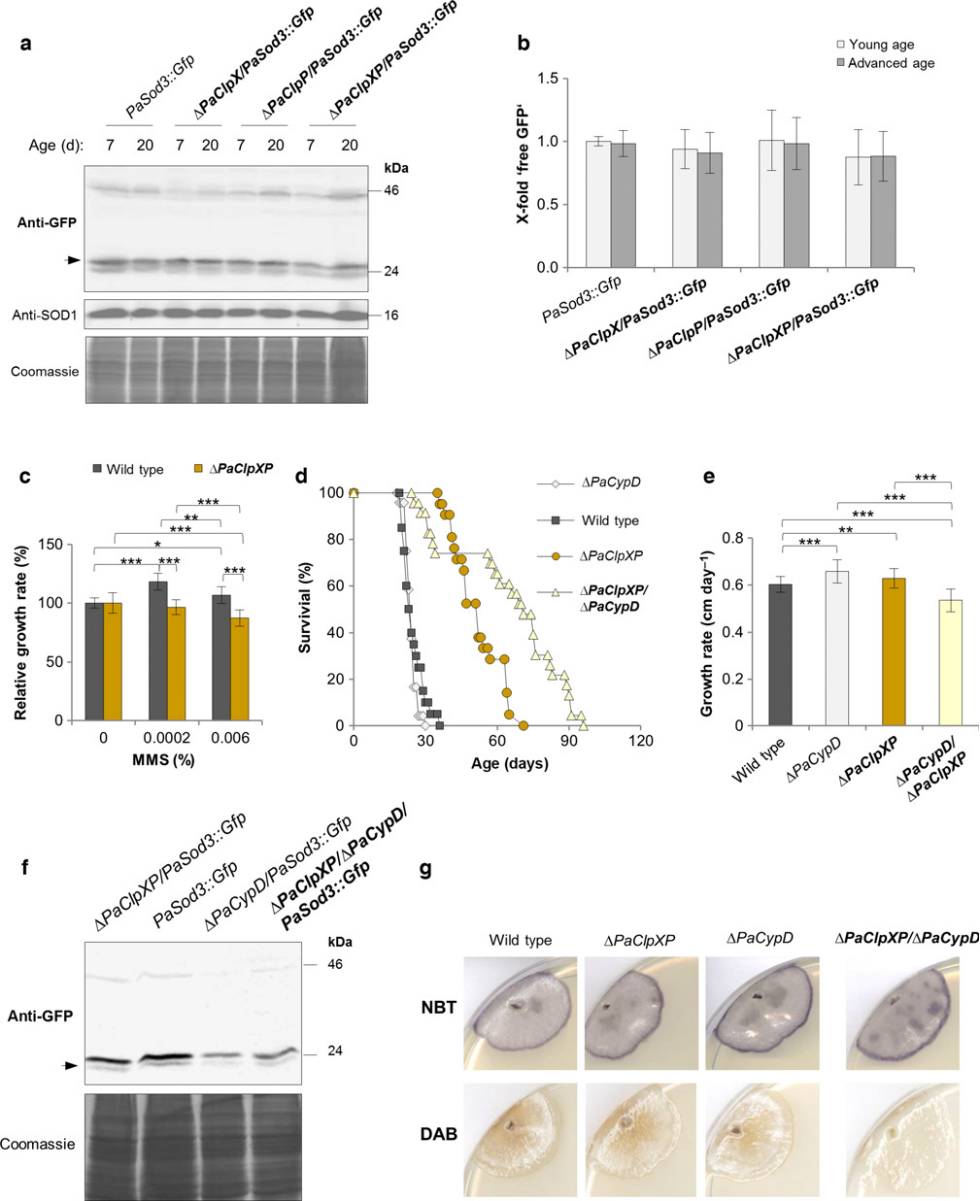
It is healthy to preserve mitochondria from degradation by mitophagy in the different *PaClpXP* deletion strains. (a) Monitoring mitophagy by western blot analysis of 7‐ and 20‐day‐old *PaSod3::Gfp* compared to *∆PaClpX*/*PaSod3::Gfp,* ∆*PaClpP*/*PaSod3::Gfp,* and ∆*PaClpXP/PaSod3::Gfp*. (b) Quantification of ‘free GFP’ protein levels of 7 (*n* = 3)‐ and 20 (*n* = 4)‐day‐old *PaSod3::Gfp* vs. ∆*PaClpX*/*PaSod3::Gfp, ∆PaClpP/PaSod3::Gfp, and ∆PaClpXP/PaSod3::Gfp* normalized to the level of PaSOD1. Protein abundance in 7‐day‐old *PaSod3::Gfp* was set to 1. Error bars correspond to the standard deviation. *P*‐values were determined by Student's *t‐*test. (c) Relative growth rates of MMS‐treated wild type (*n* = 16) and *∆PaClpXP* (*n* = 17) compared to the untreated controls. *P*‐values were determined between the wild type and the mutant and between each strain and MMS condition by two‐tailed Mann–Whitney–Wilcoxon *U*‐test. Error bars correspond to the standard deviation. (d) Survival curves of the wild type (*n* = 20), *∆PaClpXP* (*n* = 21), *∆PaCypD* (*n* = 23), and *∆PaClpXP/∆PaCypD* (*n* = 23). *P*‐values were determined between wild type and *∆PaClpXP* (*P *<* *0.001)*,* wild type and ∆*PaCypD* (*P *>* *0.05), ∆*PaClpXP* and ∆*PaCypD* (*P *<* *0.001), between ∆*PaClpXP/∆PaCypD* and ∆*PaClpXP* (*P *<* *0.05), *∆PaClpXP/∆PaCypD* and wild type (*P *<* *0.001), respectively, ∆*PaClpXP/∆PaCypD* and *∆PaCypD* (*P *<* *0.001) by two‐tailed Mann–Whitney *U*‐test. (e) Growth rate of the wild type (*n* = 20), *∆PaClpXP* (*n* = 22), *∆PaCypD* (*n* = 24), and *∆PaClpXP*/*∆PaCypD* (*n* = 26). Error bars correspond to the standard deviation, and *P*‐values were determined by two‐tailed Mann–Whitney–Wilcoxon *U*‐test. (f) Monitoring mitophagy by western blot analysis of *PaSod3::Gfp* compared to ∆*PaClpXP/PaSod3::Gfp, ∆PaCypD/PaSod3::Gfp,* and *∆PaClpXP/∆PaCypD/PaSod3::Gfp*. The Coomassie‐stained gel serves as a loading control. (g) Determination of superoxide and hydrogen peroxide in 7‐day‐old wild type, *∆PaClpXP, ∆PaCypD,* and *∆PaClpXP/∆PaCypD* strains by NBT and DAB staining. (c, e) *= *P*< 0.05, **= *P*< 0.01, *** *P*< 0.001.

In a parallel study, we demonstrated that mitophagy is upregulated during aging in a *PaSod3* deletion mutant of *P. anserina* and compensates the loss of this mitochondrial quality control component (Knuppertz *et al*., 2017). We therefore tested whether induction of mitophagy plays also a role in the different *PaClpXP* deletion mutants. As the microscopy analysis of autophagosomes does only provide evidence for the initiation of autophagy but can neither discriminate between nonselective and selective autophagy, we performed a biochemical analysis (Meiling‐Wesse *et al*., 2002; Kanki *et al*., 2009). In this approach, the fate of a reporter protein fused to GFP is followed. Upon degradation of a reporter protein via autophagy, the GFP part of the fusion protein, which remains stable in the vacuole, can be detected as ‘free GFP’ by western blot analysis. We used strains expressing a *PaSod3::Gfp* fusion gene encoding a mitochondrial protein as a marker for mitophagy (Fig. S1A). Although mitochondrial function is compromised in the analyzed *PaClpXP* mutants, and in marked contrast to what we found in a *PaSod3* deletion mutant (Knuppertz *et al*., 2017), there is no difference in mitophagy levels in wild type and *PaClpXP* deletion mutants, neither at young nor at advanced age (Fig. 2a,b). We verified this result using a previously generated *PaSOD3* ^*H26L*^ *::Gfp* mitochondrial reporter strain (Fig. S1B; Knuppertz *et al*., 2017), in which PaSOD3 is enzymatically inactive (Figs 2a,b and S3). Interestingly, in total protein extracts, the PaSOD3^H26L^::GFP full‐length protein is underrepresented and only detectable when large amounts of protein are used in western blot analysis. However, as demonstrated in previous studies, the full‐length fusion protein is clearly visible in mitochondrial extracts (Knuppertz *et al*., 2017) and the ‘free GFP’ observed in the different strains results from the degradation of the fusion protein via autophagy/mitophagy (Knuppertz *et al*., 2014, 2017).

To exclude the possibility that mitophagy is defective in the different *PaClpXP* deletion strains*,* we attempted to induce mitophagy in the different *PaClpXP* deletion mutants. After testing a number of different components and conditions (i.e., FCCP, nitrogen starvation, rotenone), we identified the alkylating agent and carcinogen methyl methanesulfonate (MMS) as an autophagy‐ and mitophagy‐inducing compound in *P. anserina* wild type (Fig. S4A–E). MMS treatment was found to increase ‘free GFP’ in the different *∆PaClpXP* mutants which express the mitophagy marker gene *PaSod3* ^*H26L*^ *::Gfp* (Fig. S3C). These data clearly demonstrate that the general capacity to induce mitophagy is not affected in strains ablated for the different PaCLPXP components. Interestingly, relative growth rate of *∆PaClpXP* under MMS treatment is significantly reduced when compared to MMS‐treated wild type, suggesting that the induction of mitophagy negatively affects vegetative growth of the *∆PaClpXP* strain (Fig. 2c).

Next, we tested the impact of lowering mitophagy beyond the basic levels found in the *∆PaClpXP* mutant. Therefore, we combined the *PaClpXP* deletion with the deletion of *PaCypD*, a gene coding for the mitochondrial peptidyl prolyl‐cis, trans‐isomerase cyclophilin D (CYPD) known to be involved in the regulation of mitochondrial permeability transition (mPT) regulation, programmed cell death (Brust *et al*., 2010), and mitophagy (Carreira *et al*., 2010; Kramer *et al*., 2016). In the latter mutant, mitophagy levels are lower than the basal levels normally observed in *P. anserina* strains (Kramer *et al*., 2016). Compared to the *PaClpXP* deletion strain, the *∆PaClpXP/∆PaCypD* mutant (Fig. S1C) is characterized by an increased lifespan (Fig. 2d), demonstrating that lifespan extension in *∆PaClpXP* is thus not dependent on the induction of mitophagy. Moreover, as indicated by the increased lifespan, in this mutant, it appears to be advantageous to protect mitochondria from degradation via mitophagy. However, this protection goes along with a slight reduction in growth rate (Fig. 2e). Supporting this hypothesis, a comparative western blot analysis of total protein extracts demonstrates that mitophagy is reduced in a *∆PaClpXP/∆PaCypD/PaSod3::Gfp* mutant compared to *PaSod3::Gfp* and *∆PaClpXP/PaSod3::Gfp* (Fig. 2f). This reduction is similar to that in the previously reported ∆*PaCypD/PaSod3::Gfp* strain (Kramer *et al*., 2016). Interestingly, we found no obvious differences in the amount of superoxide but a slight decrease in hydrogen peroxide levels in a *∆PaClpXP/∆PaCypD* mutant compared to the wild type and the single deletion mutants that probably could be co‐responsible for the healthier phenotype of this mutant (Fig. 2g).

### Nonselective general autophagy is increased and compensates for the defects in the energy metabolism of *PaClpXP* deletion mutants

**Figure 3:**
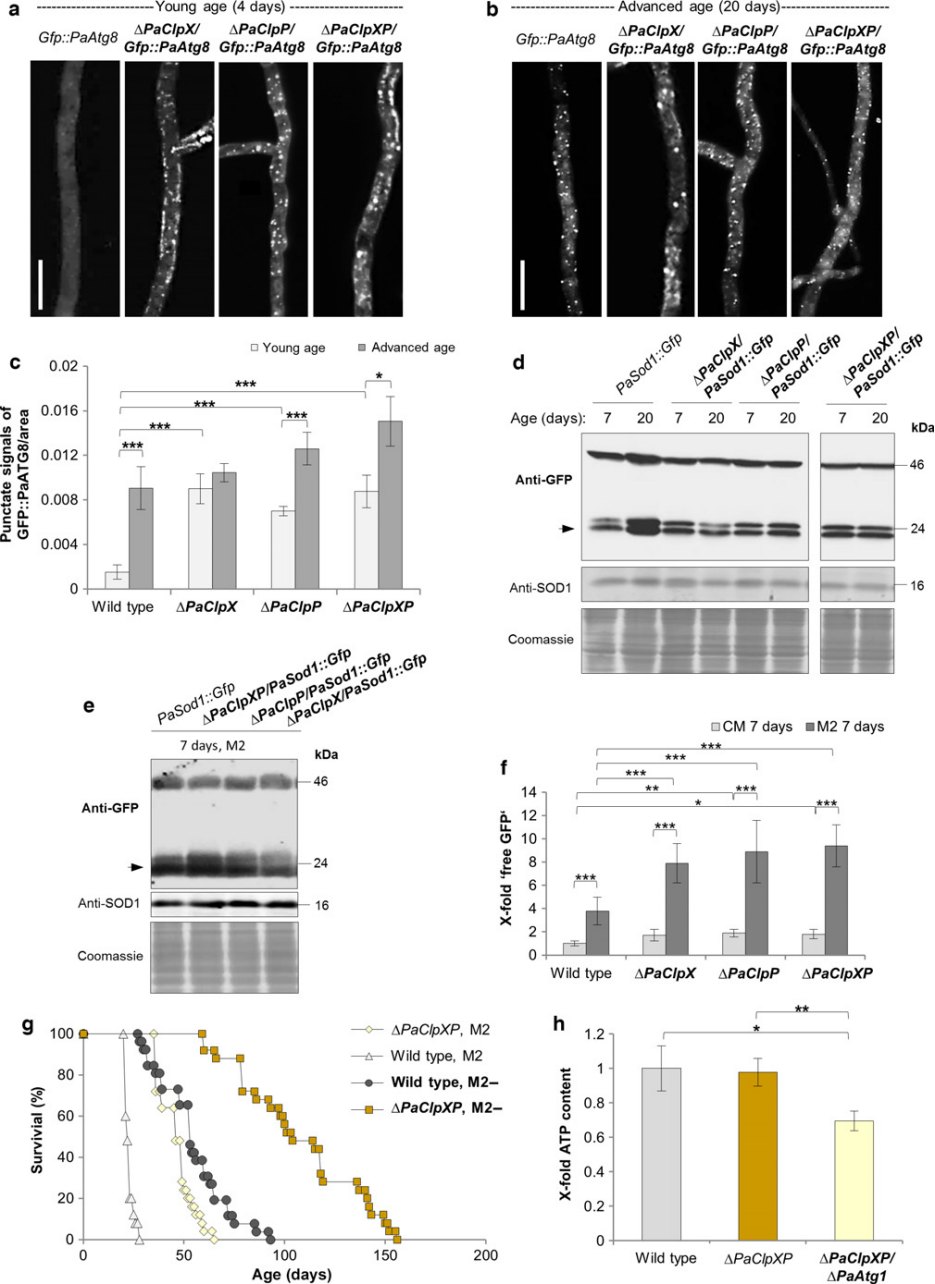
Autophagy is induced in a starvation‐like response of *PaClpXP* mutant strains. LSFM of hyphae from 4 (a)‐ and 20 (b)‐day‐old wild type and *∆PaClpX*,* ∆PaClpP,* and *∆PaClpXP* strains expressing *Gfp::PaAtg8*. (c) Quantification of autophagosomes of 4‐ and 20‐day‐old wild type and *∆PaClpX*,* ∆PaClpP,* and *∆PaClpXP* strains expressing *Gfp::PaAtg8* (*n* = 10). *P*‐values were determined between 4‐ and 20‐day‐old strains and between wild type and mutant of the same age. Error bars correspond to the standard error. *P*‐values were determined by two‐tailed Mann–Whitney–Wilcoxon *U*‐test. (d) Monitoring autophagy by western blot analysis of 7‐ and 20–day‐old *PaSod1::Gfp* compared to *∆PaClpX/PaSod1::Gfp, ∆PaClpP/PaSod1::Gfp,* and *∆PaClpXP/PaSod1::Gfp* cultivated on CM medium. (e) Monitoring autophagy by western blot analysis of 7‐day‐old *PaSod1::Gfp* compared to *∆PaClpX/PaSod1::Gfp, ∆PaClpP/PaSod1::Gfp,* and *∆PaClpXP/PaSod1::Gfp* cultivated on M2 medium. (f) Quantification of ‘free GFP’ protein levels of 7 (*n* = 4)‐day‐old *PaSod1::Gfp* vs. *∆PaClpX/PaSod1::Gfp, ∆PaClpP/PaSod1::Gfp, and ∆PaClpXP/PaSod1::Gfp* cultivated on CM or M2 medium normalized to the level of PaSOD1. Protein abundance in 7‐day‐old *PaSod1::Gfp* was set to 1. Error bars correspond to the standard deviation. *P*‐values were determined by Student's *t*‐test. (g) Survival curves of the wild type (*n* = 25) and *∆PaClpXP* (*n* = 25) cultivated on M2 vs. M2‐. *P*‐values were determined between wild type and *∆PaClpXP* on M2 (*P *<* *0.001), wild type and *∆PaClpXP* on M2‐ (*P *<* *0.001), wild type on M2 and M2‐ (*P *<* *0.001), and *∆PaClpXP* on M2 and M2‐ (*P *<* *0.001) by two‐tailed Mann–Whitney–Wilcoxon *U*‐test. (h) ATP levels of 5‐day‐old wild type, *∆PaClpXP,* and *∆PaClpXP/∆PaAtg1* (three biological replicates with three technical replicates) were measured by a luminescence‐based assay. Error bars correspond to the standard deviation, and *P*‐values were determined with paired Student's *t‐*test. (c, f, h) *= *P*< 0.05, **= *P*< 0.01, *** *P*< 0.001.

Next, we set out to elucidate the mechanistic basis of the observed lifespan extension in the different *PaClpXP* deletion mutants. From previous work, we know that autophagy is active as a longevity‐assurance mechanism that is induced during aging (Knuppertz *et al*., 2014). Moreover, we found that mild stress can lead to a beneficial hormetic response leading to an increased lifespan (Knuppertz *et al*., 2017). To test such a role of autophagy in the *PaClpXP* mutants, we quantified the abundance of autophagosomes microscopically in wild type, *∆PaClpX*,* ∆PaClpP,* and *∆PaClpXP* strains which express the *Gfp::PaAtg8* fusion gene encoding the GFP‐labeled autophagosomal marker PaATG8 (Fig. S1D). Significantly, fluorescence microscopy revealed a high number of autophagosomes in all three *PaClpXP* deletion mutants already at juvenile age when autophagosomes are almost absent from wild type cells (Fig. 3a,c). At advanced age, this number strongly increases in the wild type but only slightly in the *PaClpXP* deletion mutants (Fig. 3b,c).

To analyze whether the identified autophagosomes deliver their cargo to vacuoles where it is subsequently degraded, we analyzed the fate of the cytoplasmic PaSOD1::GFP fusion reporter protein biochemically in wild type and the different *PaClpXP* deletion strains (Fig. S1E). While ‘free GFP’ was found to be strongly increased during aging of the wild type grown in complete (CM) liquid medium, autophagy in the *PaClpXP* mutants was slightly increased in 7‐day‐old cultures compared to the wild type of the same age. However, there was no increase observed in 20‐day‐old strains (Fig. 3d,f), verifying that that 20‐day‐old strains of this long‐lived mutant are not in the same biological late state of aging as the wild type.

Autophagy is known to be important for balancing energy sources at critical times in development and in response to nutrient availability. Concordantly, when we changed cultivation conditions and grew strains on M2 solid medium, a minimal medium that we used for the microscopic analysis and lifespan experiments, autophagy was strongly induced in the wild type. Moreover, autophagy in the different *PaClpXP* deletion mutants was significantly higher on M2 compared to the corresponding wild type control (Fig. 3e,f). These results are in accordance with data from the microscopic analysis (Fig. 3a–c).

Subsequently, we investigated the impact of starvation on lifespan of the *∆PaClpXP* mutant in more detail. We determined the lifespan of both the wild type and the *∆PaClpXP* strain on standard M2 minimal medium and on M2 depleted for nitrogen and glucose (M2‐) (Fig. 3g). Interestingly, the mutant strain lives much longer on M2‐ than on M2 medium, suggesting that the applied harsh nutritional starvation is beneficial for the mutant most likely via a stronger induction of autophagy. Notably, lifespan of the wild type on M2‐ medium is increased, similar to the lifespan of *∆PaClpXP* on M2 medium (Fig. 3g).

After the demonstration of the induction of general autophagy in the *PaClpXP* deletion strains and the effects of nutritional starvation on lifespan, we next investigated whether autophagy contributes to energy conservation and ATP content in the *PaClpXP* double mutant. We measured ATP content of the *∆PaClpXP/∆PaAtg1* strain in which autophagy is blocked and compared it to the *PaClpXP* deletion strain and the wild type. Strikingly, we found that the wild type‐like ATP content of the *∆PaClpXP* strain depends on a functional autophagy machinery (Fig. 3h).

To test whether the *∆PaClpXP/∆PaAtg1* mutant is characterized by a more profound alteration in the amount of mitochondrial respiratory chain complexes than the *∆PaClpXP* mutant, thus explaining the consequent changes in ATP levels upon block of autophagy, we performed an additional BN‐PAGE experiment. We used mitochondrial extracts of 7‐day‐old wild type, *∆PaClpXP*/*∆PaAtg1* and *∆PaAtg1* strains. Interestingly, compared to wild type, we found no obvious differences in the amount of mitochondrial respiratory chain complexes in the autophagy‐deficient *PaAtg1* deletion mutant (Fig. S5). For the *∆PaClpXP/∆PaAtg1* mutant strain, we found the same characteristic differences in the protein amount of mitochondrial respiratory chain complexes as in the *PaClpXP* deletion mutant (Fig. 1d and S5). These results indicate that the detected impairments in mitochondrial respiration are caused via the loss of the PaCLPXP protease and not upon a modulated autophagy rate in this mutant. This lack of PaCLPXP activity in *P. anserina* is effectively compensated by autophagy, acting as a prosurvival pathway via the conservation of the cellular energy status.

### The healthy phenotype of *PaClpXP* deletion strains depends on functional autophagy

**Figure 4:**
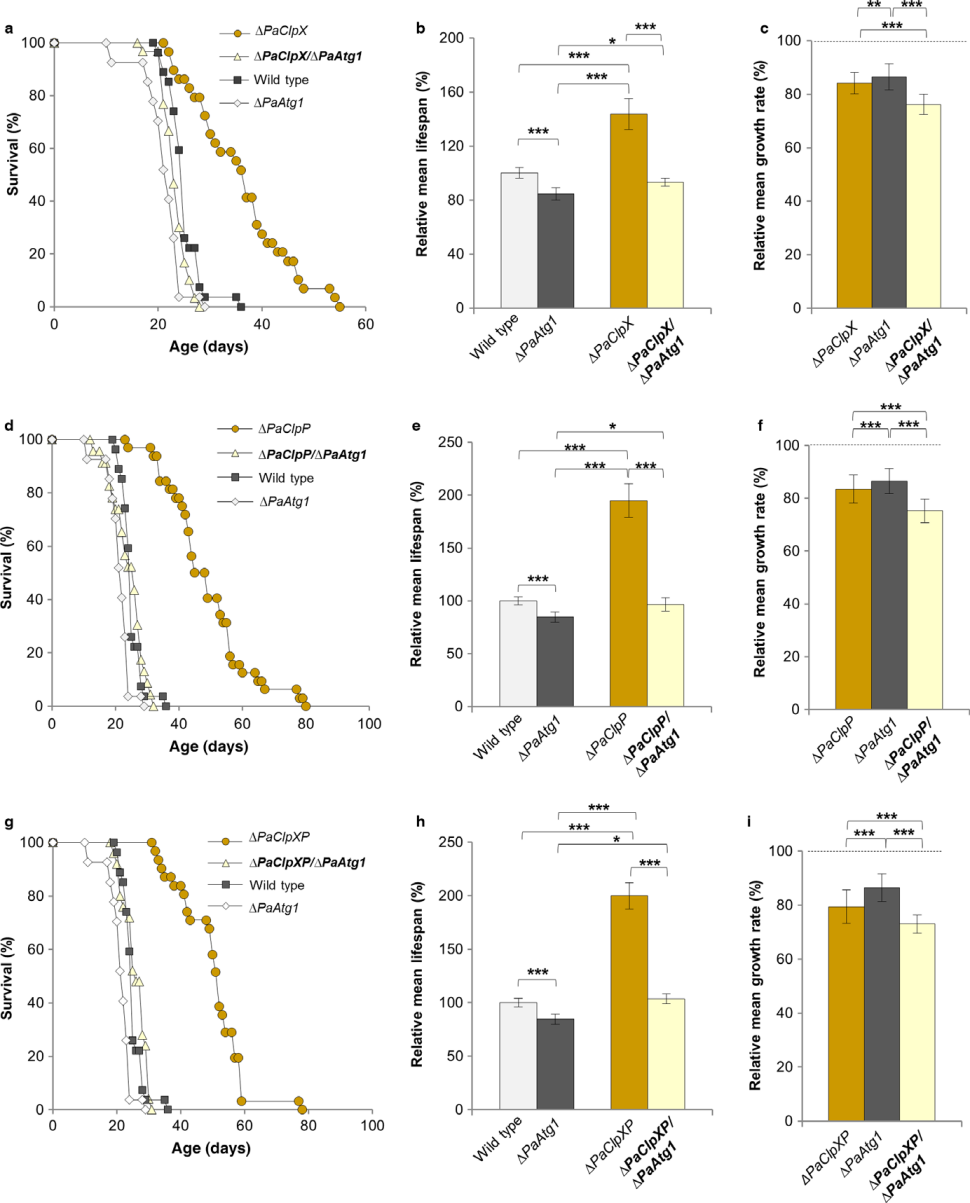
Autophagy is responsible for the healthy phenotype of *∆PaClpXP* mutants. (a) Survival curves of the wild type (*n* = 27), *∆PaAtg1* (*n* = 27; *P *<* *0.001), *∆PaClpX* (*n* = 29), and ∆*PaClpX/∆PaAtg1* (*n* = 30; *P *<* *0.001). (b) Relative mean lifespan of *∆PaAtg1* (*n* = 27), *∆PaClpX* (*n* = 29), and ∆*PaClpX/∆PaAtg1* (*n* = 30) resulting from the comparison of the mean lifespan of each strain with the mean lifespan of the wild type (*n* = 27, set to 100%). (c) Relative mean growth rates of *∆PaAtg1* (*n* = 27), *∆PaClpX* (*n* = 29), and ∆*PaClpX*/*∆PaAtg1* (*n* = 30) derived from the comparison of the mean growth rate of each strain with the mean growth rate of the wild type (*n* = 27, set to 100%). (d) Survival curves of the wild type (*n* = 27), *∆PaAtg1* (*n* = 27; *P *<* *0.001), *∆PaClpP* (*n* = 32), and ∆*PaClpP*/*∆PaAtg1* (*n* = 23; *P *<* *0.001). (e) Relative mean lifespan of *∆PaAtg1* (*n* = 27), *∆PaClpP* (*n* = 32), and ∆*PaClpP*/*∆PaAtg1* (*n* = 23) resulting from the comparison of the mean lifespan of each strain with the mean lifespan of the wild type (*n* = 27, set to 100%). (f) Relative mean growth rates of *∆PaAtg1* (*n* = 27), *∆PaClpP* (*n* = 32), and ∆*PaClpP/∆PaAtg1* (*n* = 23) derived from the comparison of the mean growth rate of each strain with the mean growth rate of the wild type (*n* = 27, set to 100%). (g) Survival curves of the wild type (*n* = 27), *∆PaAtg1* (*n* = 27; *P < *0.001), *∆PaClpXP* (*n* = 31), and ∆*PaClpXP*/*∆PaAtg1* (*n* = 25; *P *<* *0.001). (h) Relative mean lifespan of *∆PaAtg1* (*n* = 27), *∆PaClpXP* (*n* = 31), and ∆*PaClpXP*/*∆PaAtg1* (*n* = 25) resulting from the comparison of the mean lifespan of each strain with the mean lifespan of the wild type (*n* = 27, set to 100%). (i) Relative mean growth rates of *∆PaAtg1* (*n* = 27), *∆PaClpXP* (*n* = 31), and ∆*PaClpXP/∆PaAtg1* (*n* = 25) derived from the comparison of the mean growth rate of each strain with the mean growth rate of the wild type (*n* = 27, set to 100%). (b, c, e, f, h, i) Error bars correspond to the standard error, and *P*‐values were determined by two‐tailed Mann–Whitney–Wilcoxon *U*‐test. *= *P*< 0.05, **= *P*< 0.01, *** *P*< 0.001.

Next, we investigated whether the healthy phenotype of the *PaClpXP* deletion mutant depends on functional autophagy and therefore generated *ΔPaClpP/ΔPaAtg1, ΔPaClpX/ΔPaAtg1,* and *ΔPaClpXP/ΔPaAtg1* mutants, in which autophagy is completely blocked (Fig. S1F). Strikingly, in the double mutants, the longevity phenotype of *∆PaClpX*, Δ*PaClpP,* and *∆PaClpXP* is reverted to wild type level (Fig. 4a,b,d,e,g,h). In addition, the growth rate is reduced in all mutants lacking ATG1 compared to the single deletion strains and the wild type (Fig. 4c,f,i). As lifespan and growth rate are decreased by the loss of autophagy, these data demonstrate that the longevity phenotype of the mutants, in which components of the PaCLPXP complex are ablated, depends on a functional autophagy apparatus and the induction of autophagy.

### Loss of PaIAP causes a lifespan prolonging phenotype that is unaffected by autophagy

**Figure 5:**
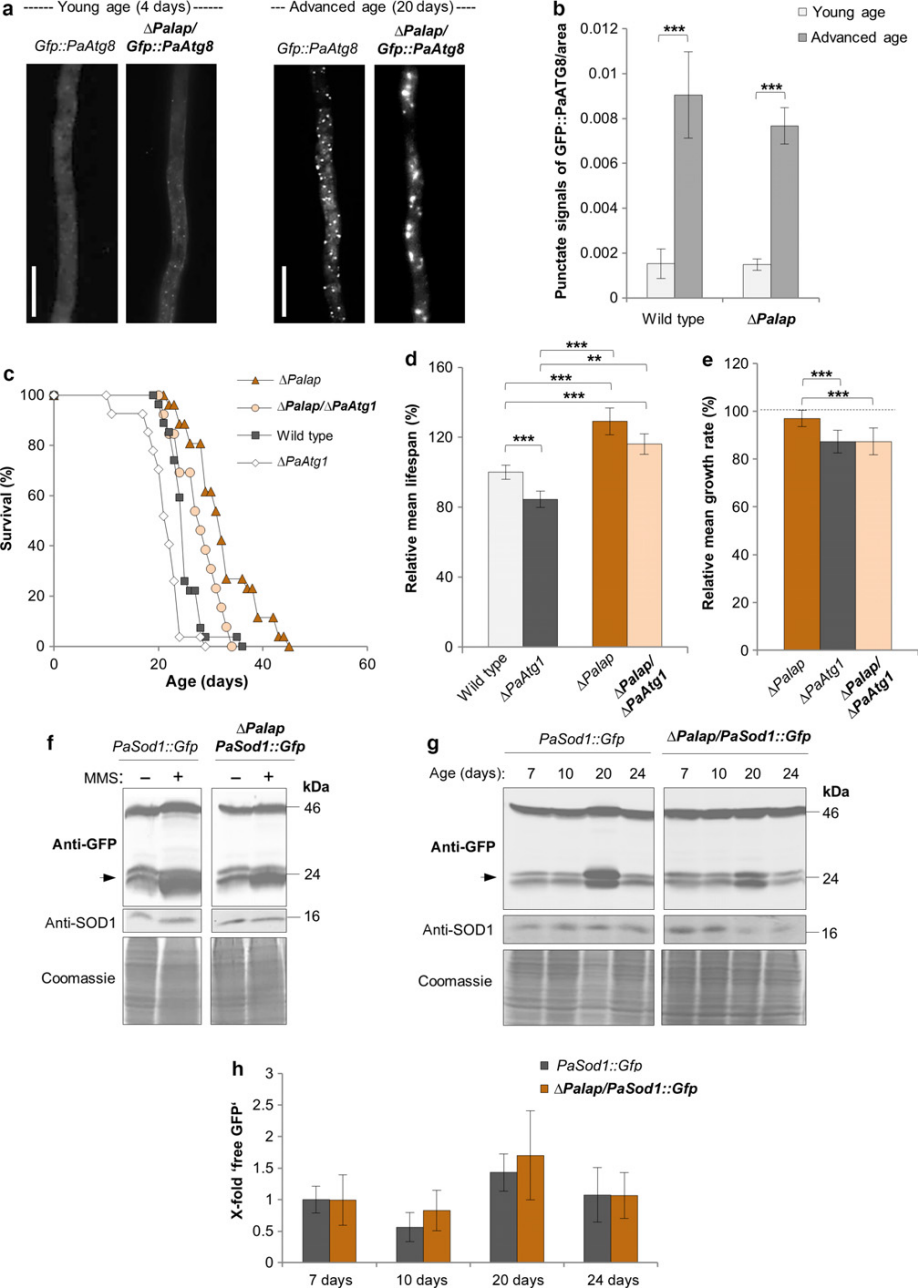
The healthy phenotype of *∆PaIap* is not autophagy dependent. (a). LSFM of hyphae from 4‐ and 20–day‐old wild type and *∆PaIap* strains expressing *Gfp::PaAtg8*. (b) Quantification of autophagosomes of 4‐ and 20‐day‐old wild type and *∆PaIap* strains expressing *Gfp::PaAtg8* (*n* = 10). *P*‐values were determined between 4‐ and 20‐day‐old strains and between wild type and mutant of the same age. Error bars correspond to the standard error. (c) Survival curves of the wild type (*n* = 27), *∆PaAtg1* (*n* = 27; *P *<* *0.001), *∆PaIap* (*n* = 46), and ∆*PaIap*/*∆PaAtg1* (*n* = 38; *P *<* *0.01). (d) Relative mean lifespan of *∆PaAtg1* (*n* = 27), *∆PaIap* (*n* = 46), and ∆*PaIap*/*∆PaAtg1* (*n* = 38) resulting from the comparison of the mean lifespan of each strain with the mean lifespan of the wild type (*n* = 27, set to 100%). (e) Relative mean growth rates of *∆PaAtg1* (*n* = 27), *∆PaIap* (*n* = 46), and ∆*PaIap*/*∆PaAtg1* (*n* = 38) derived from the comparison of the mean growth rate of each strain with the mean growth rate of the wild type (*n* = 27, set to 100%). (f) Monitoring mitophagy during MMS treatment (0.09% for the last 5 h of cultivation) by western blot analysis of 7‐day‐old *PaSod1::Gfp* compared to ∆*PaIap/PaSod1::Gfp*. (g) Monitoring autophagy by western blot analysis of 7‐day, 10‐day, 20‐day, and 24‐day‐old *PaSod1::Gfp* compared to ∆*PaIap*/*PaSod1::Gfp*. (h) Quantification of ‘free GFP’ protein levels of 7‐day (*n* = 4), 10‐day (*n* = 4), 20‐day (*n* = 4), and 24‐day (*n* = 4)‐old *PaSod1::Gfp* vs. ∆*PaIap*/*PaSod1::Gfp* normalized to the level of PaSOD1. Protein abundance in 7–day‐old *PaSod1::Gfp* was set to 1. Error bars correspond to the standard deviation. *P*‐values were determined by Student's *t‐*test. (b, d, e) *P*‐values were determined by two‐tailed Mann‐Whitney‐Wilcoxon U‐test (*= *P*< 0.05, **= *P*< 0.01, *** *P*< 0.001).

After the demonstration of the induction of autophagy in mutants affected in one component of the mitochondrial proteolysis system, we next asked the question of whether or not such a response is general or whether it is specifically depending on the kind of perturbation. As an example of another mitochondrial protease, we chose PaIAP, an *i*‐AAA protease located in the inner mitochondrial membrane. Like in the different *PaClpXP* deletion strains, lifespan of the *PaIap* deletion strain is strongly increased (Weil *et al*., 2011). To test whether autophagy is linked to this phenotype, we measured autophagosome abundance in a *∆PaIap/Gfp::PaAtg8* strain (Fig.S1D). Strikingly, compared to wild type, we did not observe significant differences in the amount of autophagosomes either in the young or in the advanced age (Fig. 5a,b). In addition, as demonstrated by the unaffected lifespan of a *∆PaIap/∆PaAtg1* double mutant (Fig. S1F), the increase in lifespan of *∆PaIap* is not dependent on a functional autophagy machinery (Fig. 5c,d). Relative growth rate is slightly reduced in the double mutant, identifying that the loss of *PaAtg1* affects vital functions in *∆PaIap* (Fig. 5e). MMS treatment of *PaSod1::Gfp* compared to *∆PaIap/PaSod1::Gfp* (Fig. S1E) revealed that autophagy is inducible and not defective in *∆PaIap* (Fig. 5f). In addition, autophagy is unaltered during aging of the ∆*PaIap/PaSod1::Gfp* strain compared to the wild type control (Fig. 5g,h). Overall, these initial cellular characteristics of *∆PaIap* compared to those of *∆PaClpXP* suggest that mitochondrial proteases have specific functions beside their role in degradation of damaged proteins. Compromising their function does not induce one general but different, specific responses.

## Discussion

Maintenance of cellular homeostasis is a key to keep biological systems functional and healthy. Impairments lead to aging and the development of disorders and severe diseases. A number of different surveillance pathways are active to balance cellular homeostasis over the lifetime of biological systems. These pathways may interact with each other allowing the compensation of impairments in one pathway or after overwhelming its capacity. The details about the underlying cross talks and their mechanistic basis are only initially elucidated.

Here, we reported novel data identifying autophagy, the vacuolar degradation of cytoplasmic components, as a mechanism involved in the compensation of impairments in mitochondrial protein quality control in a mutant of the fungal aging model *P. anserina* in which PaCLPP has been ablated and the lifespan is unexpectedly increased.

CLPP is the proteolytic component of a mitochondrial matrix protein complex consisting of CLPP and a chaperone, CLPX, responsible for delivering substrates to the peptidase for degradation. While the complex, or derivatives of it, is found in prokaryotes and mitochondria and plastids of most eukaryotes, it is remarkable that CLPP is missing in yeast. Certainly because of this lack, the biological role of CLPXP is only initially understood.

Importantly, in our study, we found that nonselective autophagy is upregulated in the *∆PaClpP* and the *∆PaClpX* single mutants and the *PaClpXP* double deletion mutant. Moreover, we demonstrated that lifespan extension of this mutant depends on a functional autophagy machinery. Certainly, more data are required to unravel the details about which components of this machinery lead to healthspan extension. However, our current view of a protective role for autophagy in the *PaClpXP* deletion strain is in concordance with previous results that uncovered general autophagy as a longevity‐assurance mechanism in *P. anserina* (Knuppertz *et al*., 2014). In wild type aging of this aging model, general autophagy becomes upregulated in the later phases of life when the mitochondrial energy metabolism is severely impaired due to the accumulation of mitochondrial dysfunction (Osiewacz & Kimpel, 1999; Borghouts *et al*., 2001). Significantly, the deletion of genes coding for the two components of the PaCLPXP led to the constitutive induction of general autophagy. The identification of CLPXP substrates and interaction partners in *P. anserina* and in mammalian cells suggests that this protease is specifically involved in the control of the mitochondrial energy metabolism (Cole *et al*., 2015; Fischer *et al*., 2015; Szczepanowska *et al*., 2016). It thus appears that both during wild type aging and as a result of PaCLPXP ablation, the resulting perturbation of the energy metabolism is the trigger for autophagy induction. In this process, sensing of the cellular nutrient status by adenosine monophosphate‐activated protein kinase (AMPK) and subsequent signaling to conserve and generate ATP by initiating different responses such as increasing glucose uptake, glycolysis, fatty acid oxidation, halting protein synthesis, and the induction in alternative oxidation (Borghouts *et al*., 2001; Jones *et al*., 2005; Kelekar, 2005; Shaw, 2009) may also play a role in the *PaClpXP* deletion strains but remain to be experimentally addressed in future experiments.

**Figure 6:**
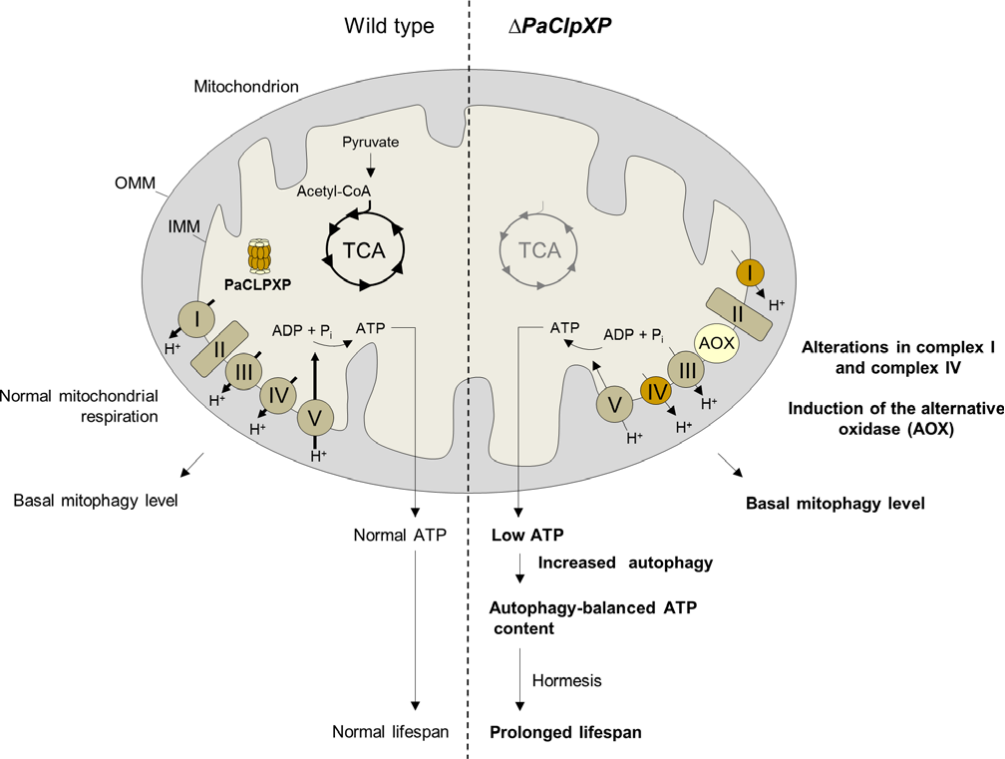
Regulation of aerobic metabolism and autophagy in the wild type compared to *∆PaClpXP*. In the wild type, NADH generated by the tricarboxylic acid cycle (TCA) is used by oxidative phosphorylation to generate ATP, and thus only basal autophagy levels occur. Because there are no limitations in mitochondrial function, mitophagy level and lifespan behave normal in *Podospora anserina* wild type. Ablation of the mitochondrial AAA+ protease PaCLPXP leads to modifications in mitochondrial respiratory (complex I, complex IV, and complex V) chain and energy metabolism. This deficiency is compensated by molecular responses leading to the induction of the alternative oxidase (AOX) and by the upregulation of unspecific general autophagy, compensating for a decreased ATP content and leading to an increased lifespan of *PaClpXP* deletion mutants as a hormetic response to mild stress. Figure is modified according to Rizzuto *et al*. (2012). OMM, outer mitochondrial membrane; IMM, inner mitochondrial membrane.

Concordantly, our current study revealed that the ablation of components of PaCLPXP indeed affects mitochondrial respiration. We found changes in mitochondrial respiratory chain complex I, IV, and V composition and an overall decreased OCR, while ATP content is unaltered compared to the wild type. Interestingly, we demonstrated that ATP content is significantly decreased in *∆PaClpXP/∆PaAtg1* when autophagy is defective (summarized in Fig. 6). These findings agree with those in yeast, where general autophagy is induced in strains in which mitochondrial function is affected by respiratory inhibitors (Deffieu *et al*., 2013). Moreover, our results are in concordance with those of previous studies on mammalian cell culture showing that the loss of autophagy reduces ATP levels (Hubbard *et al*., 2010; Tang & Rando, 2014). Along these lines, we conclude that autophagy in *PaClpXP* deletion mutants is induced as the result of decreased mitochondrial ATP generation and that the bioenergetic demands of the mutants are balanced by increased autophagy leading to a hormetic response and an increased lifespan. Such a response was recently also observed in *P. anserina* wild type, in which mitophagy was induced via the application of mild oxidative stress (Knuppertz *et al*., 2017).

In striking contrast to what we found for the different *PaClpXP* mutants, the increase in lifespan of a *PaIap* deletion mutant, in which another mitochondrial protease is ablated (Weil *et al*., 2011), is independent of autophagy. The underlying compensation mechanism in this mutant is currently unclear and remains to be unraveled. In yet another *P. anserina* mutant, we found that the induction of mitophagy efficiently compensates the ablation of the mitochondrial superoxide dismutase, a component of the ROS scavenging system (Knuppertz *et al*., 2017). In this mutant, superoxide was identified to trigger the induction of mitophagy.

Overall, our data show that impairments of components of the mitochondrial quality control network do not generally lead to the induction of general autophagy as a basic cellular compensation mechanism but to specific responses, which depend on the nature of the perturbation. The underlying specific signaling mechanisms are currently only at the beginning to be understood. They certainly hold the key to understand the basis of the different responses. Apart from this basic relevance, our work provides novel data about the role of CLPXP, which emerges to be of important relevance for aging and specific adverse effects such as Perrault syndrome or different forms of human cancer (Fischer *et al*., 2013; Gispert *et al*., 2013; Jenkinson *et al*., 2013; Cole *et al*., 2015; Deepa *et al*., 2016; Seo *et al*., 2016). Studies using the *P. anserina* model system will continue to contribute to the elucidation of the cellular functions of this component of the mitochondrial quality control network and of its interactions with other cellular surveillance pathways.

## Experimental procedures

An expanded section describing experimental procedures is available in Data S1.

### *P. anserina* strains

*Podospora anserina* wild type strain ‘s’ (Rizet, 1953), the *PaSod1::Gfp, PaSod3::Gfp* (Zintel *et al*., 2010) and *∆PaClpX*,* ∆PaClpP* (Fischer *et al*., 2013, 2015), *∆PaIap* (Weil *et al*., 2011) and *∆PaCypD* (Brust *et al*., 2010) as well as the *∆PaAtg1*,* Gfp::PaAtg8* (Knuppertz *et al*., 2014), *PaSod3* ^*H26L*^ *::Gfp* (Knuppertz *et al*., 2017) strains, and newly generated mutants were used. These are as follows: *∆PaClpP/∆PaClpX* (simplified as *∆PaClpXP),* ∆*PaClpX*/*∆PaAtg1,* **∆** *PaClpP* **/** *∆PaAtg1,* **∆** *PaClpXP* **/** *∆PaAtg1,* ∆*PaClpX*/*PaSod1::Gfp, ∆PaClpP*/*PaSod1::Gfp, ∆PaClpXP*/*PaSod1::Gfp,* ∆*PaClpX*/*PaSod3::Gfp,* ∆*PaClpP*/*PaSod3::Gfp, ∆PaClpXP/PaSod3::Gfp,* ∆*PaClp*X/*PaSod3* ^*H26L*^ *::Gfp, ∆PaClpP/PaSod3* ^*H26L*^ *::Gfp, ∆PaClpXP/PaSod3* ^*H26L::*^ *Gfp* and *∆PaClpX/Gfp::PaAtg8, ∆PaClpP/Gfp::PaAtg8* and *∆PaClpXP/Gfp::PaAtg8, ∆PaIap/PaSod1::Gfp, ∆PaIap/Gfp::PaAtg8,* ∆*PaIap*/∆*PaAtg1, and ∆PaClpXP/∆PaCypD*. Double mutants were obtained after crossing the single‐mutant strains (e.g., *∆PaClpX* with *∆PaClpP*) and selection of strains from the progeny containing both mutations (e.g., *∆PaClpXP*).

### Statistical analysis

For statistical analyses of lifespan, growth rate, oxygen consumption measurements, and autophagosome determination, two‐tailed Mann–Whitney–Wilcoxon *U*‐test was used. For the statistical analysis of protein amounts during western blot analyses and ATP measurements, we used paired Student's *t*‐test. The respective samples were compared with the appropriate wild type sample. For all analyses, the minimum level of statistical significance was set at *P *<* *0.05 (not significant different means *P *>* *0.05; significant different (*) means *P *<* *0.05; highly significant different (**) means *P *<* *0.01; very highly significant different (***) means *P *<* *0.001).

## Funding

This work was supported by grants of the Deutsche Forschungsgemeinschaft (Os75/15‐1; SFB1177) to HDO and by the LOEWE excellence initiative (project: Integrated Fungal Research) of the state of Hesse (Germany).

## Conflict of interest

None declared.

## Author contributions

HDO and LK designed this study and wrote the manuscript. LK performed the experiments. HDO supervised the study.

## Supporting information


**Fig. S1** Southern blot analyses of genomic DNA for verification of different *P. anserina* mutant strains.
**Fig. S2** Determination of the mitochondrial AOX protein amount in *∆PaClpXP* and the wild type.
**Fig. S3** Western blot analysis using the *PaSod3* ^*H26L*^ *::Gfp* mitophagy reporter strain.
**Fig. S4** Methyl methanesulfonate (MMS) as a tool to study mitophagy induction in *P. anserina*.
**Fig. S5** BN‐PAGE analysis of mitochondrial extracts from *∆PaClpXP/∆PaAtg1* and ∆*PaAtg1* compared to wild type.
**Data S1** Supporting experimental procedures.
