# Supplementary material for: Autophagy compensates impaired energy metabolism in CLPXP‐deficient Podospora anserina strains and extends healthspan
Source: Aging Cell. 2017 Apr 27;16(4):704–15. doi: 10.1111/acel.12600 (PMC5506401; doi:10.1111/acel.12600)
Supplement: Supplementary file 1 — Fig. S1 Southern blot analyses of genomic DNA for verification of different P. anserina mutant strains. Fig. S2 Determination of the mitochondrial AOX protein amount in ∆PaClpXP and the wild type. Fig. S3 Western blot analysis using the PaSod3 H26L ::Gfp mitophagy reporter strain. Fig. S4 Methyl methanesulfonate (MMS) as a tool to study mitophagy induction in P. anserina. Fig. S5 BN‐PAGE analysis of mitochondrial extracts from ∆PaClpXP/∆PaAtg1 and ∆PaAtg1 compared to wild type. Data S1 Supporting experimental procedures. [file ACEL-16-704-s001.docx]

**Supporting Information to**

Autophagy compensates impaired energy metabolism in CLPXP deficient *Podospora anserina* strains and extends healthspan

Laura Knuppertz, Heinz D. Osiewacz

**Data S1: Supporting experimental procedures**

**Cultivation of strains.** Strains were grown on standard cornmeal agar (BMM) at 27 °C under constant light (Esser, 1974). For germination of spores standard cornmeal agar (BMM) with 60 mM ammonium acetate was used and incubated at 27 °C in dark for 2 days. All strains used in this study were derived from monokaryotic ascospores (Osiewacz et al., 2013). All transgenic strains are in the genetic background of wild-type strain “s”.

**Western blot analysis.** For extraction of total protein, mycelia from different *P. anserina* strains were allowed to overgrow a cellophane foil covered M2 agar plate for 2 days at 27 °C and constant light. Subsequently, pieces of mycelia were transferred into CM-liquid medium (Osiewacz et al., 2013) grown for 3 days at 27 °C under constant light and shaking. In contrast, paraquat-untreated and treated cultures were transferred for 2 days in CM-liquid medium containing no or 60 µM paraquat (Sigma-Aldrich, 856177). Harvested mycelia were pulverized in liquid nitrogen and the protein was isolated from the powder as described (Osiewacz et al., 2013). 100-200 μg total protein extracts of *P. anserina* strains, were fractionated by two-phase SDS-PAGE (12 % separating gels) according to the standard protocol (Wittig et al., 2006). After electrophoresis, proteins were transferred to PVDF membranes (Immobilon Transfer Membranes, Merck Millipore, IPFL00010). The antibodies used in this study were described in a previous study (Knuppertz et al., 2014).

**Isolation of mitochondria.** *P. anserina* strains were grown on cellophane foil covered solid M2 agar for two days at 27 °C and constant light. After transfer of pieces from growing mycelia into CM-liquid medium, culteres were incubated for three days at 27 °C under constant light and shaking. Mitochondria of *P. anserina* cultures were subsequently isolated by differential centrifugation (Osiewacz et al., 2013).

**Mitochondrial oxygen consumption.** Determination of mitochondrial oxygen consumption was performed at 27 °C using a high-resolution respirometer (Oxygraph-2k series G, Oroboros Instruments, Innsbruck, Austria). 200 µg freshly prepared mitochondria were injected into 2 ml air saturated oxygen buffer (0.3 M sucrose, 10 mM KH_2_PO_4_, 5 mM MgCl_2_, 1 mM EDTA, 10 mM KCl, 0.1 % BSA; pH 7.2). The following substances were added serially: 20 mM pyruvate (Sigma-Aldrich, P2256) and 5 mM malate (Sigma-Aldrich, M1000), to promote complex I-dependent state 4 respiration (state 4 CI); 1.5 mM ADP (Sigma-Aldrich, A5285) was added to determine complex I-dependent state 3 respiration (state 3 CI); 10 mM succinate (Sigma-Aldrich, S2378) was used to stimulate complex I/II-dependent state 3 respiration (state 3 CI/II) and finally 1 µM rotenone (Sigma-Aldrich R8875) was added for inhibition of complex I to detect complex II-dependent state 3 respiration (state 3 CII). Data were analyzed using the DatLab 6 software from Oroboros.

For AOX and C IV OCR measurements small pieces of mycelium were transferred into the Oroboros respirometer and the oxygen consumption was analyzed in liquid CM medium. The obtained data were standardized using the dry weight of the analyzed mycelium. Beside measurements with specific inhibitors, namely KCN and SHAM (salicylhydroxamic acid), were performed. While KCN (final concentration of 1 mM) inhibits COX, SHAM (final concentration of 4 mM) inhibits the AOX.

**Blue-native polyacrylamide gels (BN-PAGE)**. BN-PAGE was performed according to Wittig et al. (2006). For preparation of each sample, 100 μg of mitochondrial protein extracts were solubilized using a digitonin (Sigma-Aldrich, D141)/ protein ratio of 3:1 (w/w). Linear gradient gels (4-13 %) overlaid with 3.5 % stacking gels were used for separation of the solubilized samples. Respiratory chain components were visualized by Coomassie blue staining and assigned as described previously (Krause et al., 2004). Complex V (CV) staining was carried out according to a modified version of the protocols from Wittig and Schägger (2005) and Suhai et al. (2009). Briefly, gels were incubated for 30 min in an ATPase-Assay dilution (270 mM glycine, 8 mM ATP (Sigma-Aldrich, A3377); 14 mM MgSO_4_, 0.2 % (w/v) Pb(NO_3_)_2_ (Merck, 107398), 35 mM Tris, pH 7.8). Subsequently, gels were shortly washed with H_2_O and then incubated in 50% methanol for 10 min to stop the reaction. Complex V monomers and dimers are visible as brown bands (here: inverted illustration shown) after a short incubation of the gel for 10 seconds in 1% ammonium sulfide.

**Determination of free ATP.** ATP levels were measured by using the ATP Bioluminescence Assay Kit (CLS II, Roche, Sigma-Aldrich: 11699695001) adapted for use in a microtiter plate format. 100 mg mycelium of each sample was put into 200 µl of ATP-Isolation buffer (100 mM NaCl, 50 mM KH_2_PO_4_, pH 6) and then immediately frozen to −80°C for 30 minutes. Samples were submersed in a boiling water bath for 15 minutes. After boiling, they were diluted in 300 µl water and smashed with glass beads (Homogenizer “Precellys24”, Peqlab) for two minutes and 5000 rpm. Subsequently, probes were again submersed in a boiling water bath for 10 minutes and finally centrifuged (10 min, 14000 rpm). Different dilutions with the supernatant were made and the assay was performed according to the manufacturer’s instructions.

**Determination of „free GFP“.** This modified experimental approach was originally developed to monitor mitophagy in yeast (Meiling-Wesse et al., 2002; Kanki et al., 2009). In this approach, in principle, the fate of a GFP fusion protein is followed. Upon degradation via autophagy, the processed GFP remains stable and can be detected by western blot analysis. Total protein extracts were subjected to western blot analysis with a GFP antibody. The corresponding strains are described in Zintel et al. (2010) and Knuppertz et al. (revised). PaSOD1::GFP is localized in the cytosol, PaSOD3::GFP and PaSOD3^H26L^::GFP in mitochondria. As a mitochondrial protein PaSOD3 is under-represented in total protein extracts of *PaSod3::Gfp and PaSod3^H26L^::Gfp* strains. Thus, only the processed GFP is visible.

**Superoxide and hydrogen peroxide release measurements.** Qualitative determination of superoxide, respectively hydrogen peroxide release from mycelia, was performed by monitoring reduction of nitroblue tetrazolium (NBT, Sigma-Aldrich, N6876) or oxidation of diaminobenzidine (DAB, Sigma-Aldrich, D-8001). *P. anserina* strains were cultivated for 3 days on M2 agar medium in the dark and 27 °C. The plates were floated with 5 ml staining solution for superoxide (contains 5 mM MOPS pH 7.6, 2.5 mM NBT) or 5 ml staining solution for hydrogen peroxide (contains 100 mM Tris/HCl pH 6.9, 2.5 mM DAB; dissolved at 60 °C for 10 min) and incubated for 30 min in the dark and 27 °C until the desired differential intensity of stain of the strains is obtained.

**Southern blot analysis.** Isolation of total DNA of *P. anserina* was carried out according to the protocol developed by Lecellier and Silar (Lecellier and Silar, 1994). DNA digestion, gel electrophoresis and Southern blotting were performed according to standard protocols. For Southern blot hybridization analysis, Digoxigenin-labeled hybridization probes (DIG DNA Labeling and Detection Kit, Roche Applied Science, 11175033910) were used according to the manufacturer’s protocol. The *PaAtg1-* , *PaAtg8*-and *Gfp*-specific probes were generated according to Knuppertz and colleagues (Knuppertz et al., 2014). As a hybridization probe specific for the *phleomycin* resistance gene (*Ble*), the 1293 bp BamHI-fragment of the plasmid pKO4 (Luce and Osiewacz, 2009) was used. The 736 bp XhoI-fragment of the plasmid pSM4 (Zintel et al., 2010) was utilized as a *hygromycin* resistance gene (*Hyg*) specific hybridization probe. The *PaClpP*- and *PaIap-* specific hybridization probe were generated according to Fischer et al. (2013). The hybridization probe specific for the *PaClpX* gene was prepared according to Fischer et al. (2015) and the *PaCypD*-specific probe was constructed as described in Brust et al. (2010).

**References**

Brust D, Daum B, Breunig C, Hamann A, Kühlbrandt W, Osiewacz HD (2010) Cyclophilin D links programmed cell death and organismal aging in *Podospora anserina*. *Aging Cell* **9**, 761-5.

Esser K (1974) *Podospora anserina*. In *Handbook of Genetics* (King RC, ed.)*.* New York: Plenum Press, pp. 531-51.

Fischer F, Langer JD, Osiewacz HD (2015) Identification of potential mitochondrial CLPXP protease interactors and substrates suggests its central role in energy metabolism. *Sci. Rep.* **5,** 18375.

Fischer F, Weil A, Hamann A, Osiewacz HD (2013) Human CLPP reverts the longevity phenotype of a fungal ClpP deletion strain. *Nat. Commun.* **4,** 1397.

Kanki T, Kang D, Klionsky DJ (2009) Monitoring mitophagy in yeast: the Om45-GFP processing assay. *Autophagy* **5,** 1186-9.

Knuppertz L, Hamann A, Pampaloni F, Stelzer E, Osiewacz HD (2014) Identification of autophagy as a longevity-assurance mechanism in the aging model *Podospora anserina*. *Autophagy* **10,** 822-34.

Knuppertz L, Warnsmann V, Hamann A, Grimm C, Osiewacz HD (2017). Stress-dependent opposing roles for mitophagy in aging of the ascomycete *Podospora anserina. Autophagy***.** DOI:10.1080/15548627.2017.1303021

Krause F, Scheckhuber CQ, Werner A, Rexroth S, Reifschneider NH, Dencher NA, Osiewacz HD (2004). Supramolecular organization of cytochrome c oxidase- and alternative oxidase-dependent respiratory chains in the filamentous fungus *Podospora anserina*. *J. Biol. Chem.* **279,** 26453-61.

Lecellier G, Silar P (1994) Rapid methods for nucleic acids extraction from Petri dish-grown mycelia. *Curr. Genet.* **25,** 122-3.

Luce K, Osiewacz HD (2009) Increasing organismal healthspan by enhancing mitochondrial protein quality control. *Nat. Cell Biol*. **11,** 852-8.

Meiling-Wesse K, Barth H, Thumm M (2002) Ccz1p/Aut11p/Cvt16p is essential for autophagy and the cvt pathway. *FEBS Lett.* **526,** 71-6.

Osiewacz HD, Hamann A, Zintel S (2013) Assessing organismal aging in the filamentous fungus *Podospora anserina*. *Methods Mol. Biol.* **965**, 439-62.

Suhai T, Heidrich NG, Dencher NA, Seelert H (2009) Highly sensitive detection of ATPase activity in native gels. *Electrophoresis* **30,** 3622-5.

Wittig I, Braun HP, Schägger H (2006) Blue native PAGE. *Nat. Protoc.* **1,** 418-28.

Wittig I, Schägger H (2005) Advantages and limitations of clear-native PAGE. *Proteomics* **5,** 4338-46.

Zintel S, Schwitalla D, Luce K, Hamann A, Osiewacz HD (2010) Increasing mitochondrial superoxide dismutase abundance leads to impairments in protein quality control and ROS scavenging systems and to lifespan shortening. *Exp. Gerontol.* **45,** 525-32.


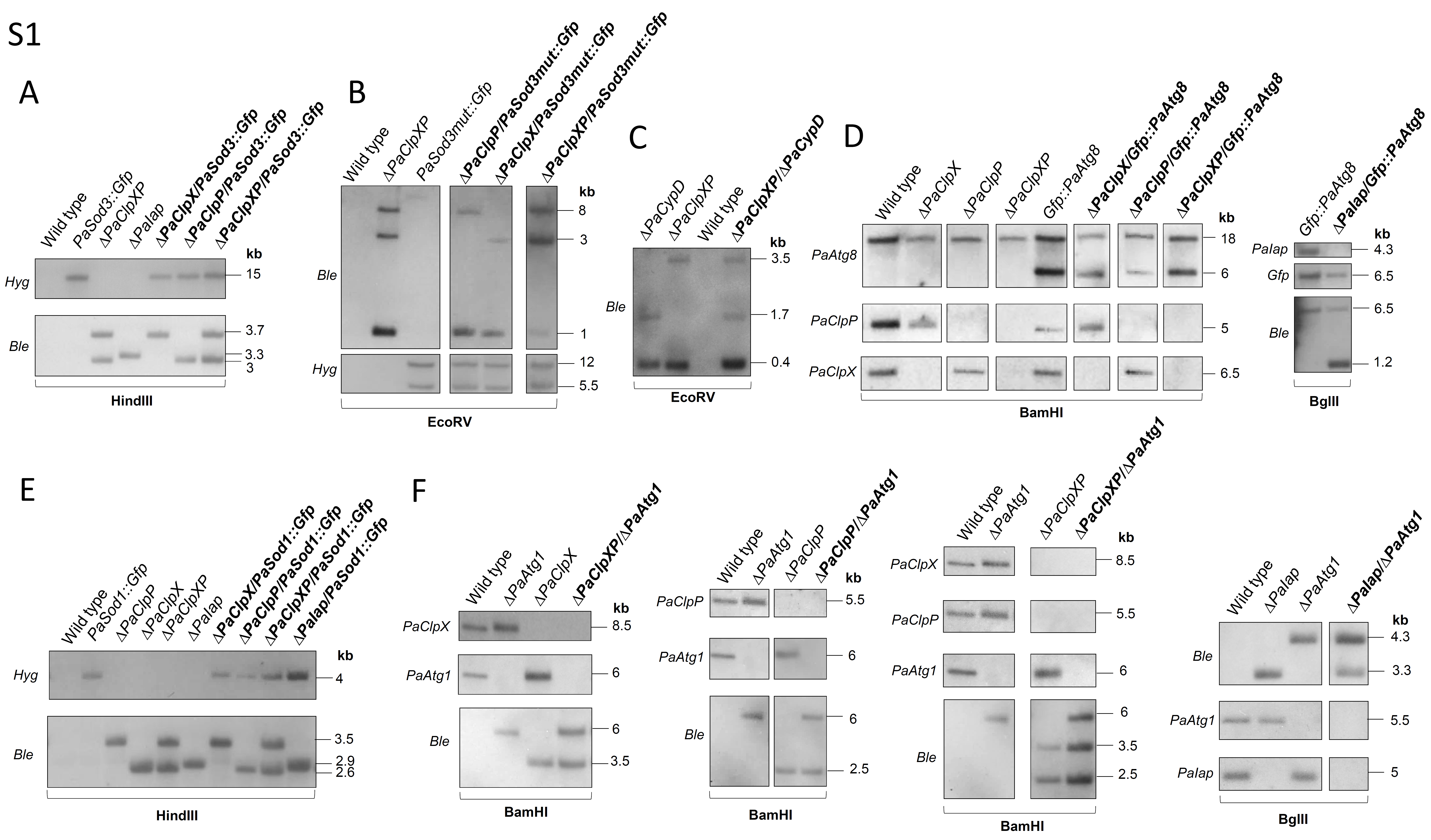
**Figure S1**

**Figure S1: Southern blot analyses of genomic DNA for verification of different *P. anserina* mutant strains. (A)** Southern blot analysis of HindIII digested genomic DNA validating the genetic constitution of different *PaSod3::Gfp* mutants and their respective control strains. *Ble-* (*phleomycin* resistance), *Hyg-* (*hygromycin* resistance) probes were used as indicated. **(B)** Southern blot analysis of EcoRV digested genomic DNA validating the genetic constitution of different *PaSod3mut::Gfp* mutants and their respective control strains. *Ble-* (*phleomycin* resistance), *Hyg-* (*hygromycin* resistance) probes were used as indicated. **(C)** Southern blot analysis of EcoRV digested genomic DNA validating the genetic constitution of *a ∆PaClpXP/∆PaCypD* triple mutant and the respective control strains. *Ble-* (*phleomycin* resistance) probe was used as indicated. **(D)** Southern blot analysis of BamHI, respectively BglII digested genomic DNA validating the genetic constitution of different *Gfp::PaAtg8* mutants and their respective control strains. *Ble-* (*phleomycin* resistance), *Gfp*, *PaClpP*, *PaClpX, PaAtg8*- and *PaIap* probes were used as indicated. **(E)** Southern blot analysis of HindIII digested genomic DNA validating the genetic constitution of different *PaSod1::Gfp* mutants and their respective control strains. *Ble-* (*phleomycin* resistance), *Hyg-* (*hygromycin* resistance) probes were used as indicated. **(F)** Southern blot analysis of BamHI digested genomic DNA validating the genetic constitution of different *∆PaAtg1* mutants and their respective control strains. *Ble-* (*phleomycin* resistance), *PaClpX, PaClpP, PaAtg1* and *PaIap* probes were used as indicated.

**Figure S2**


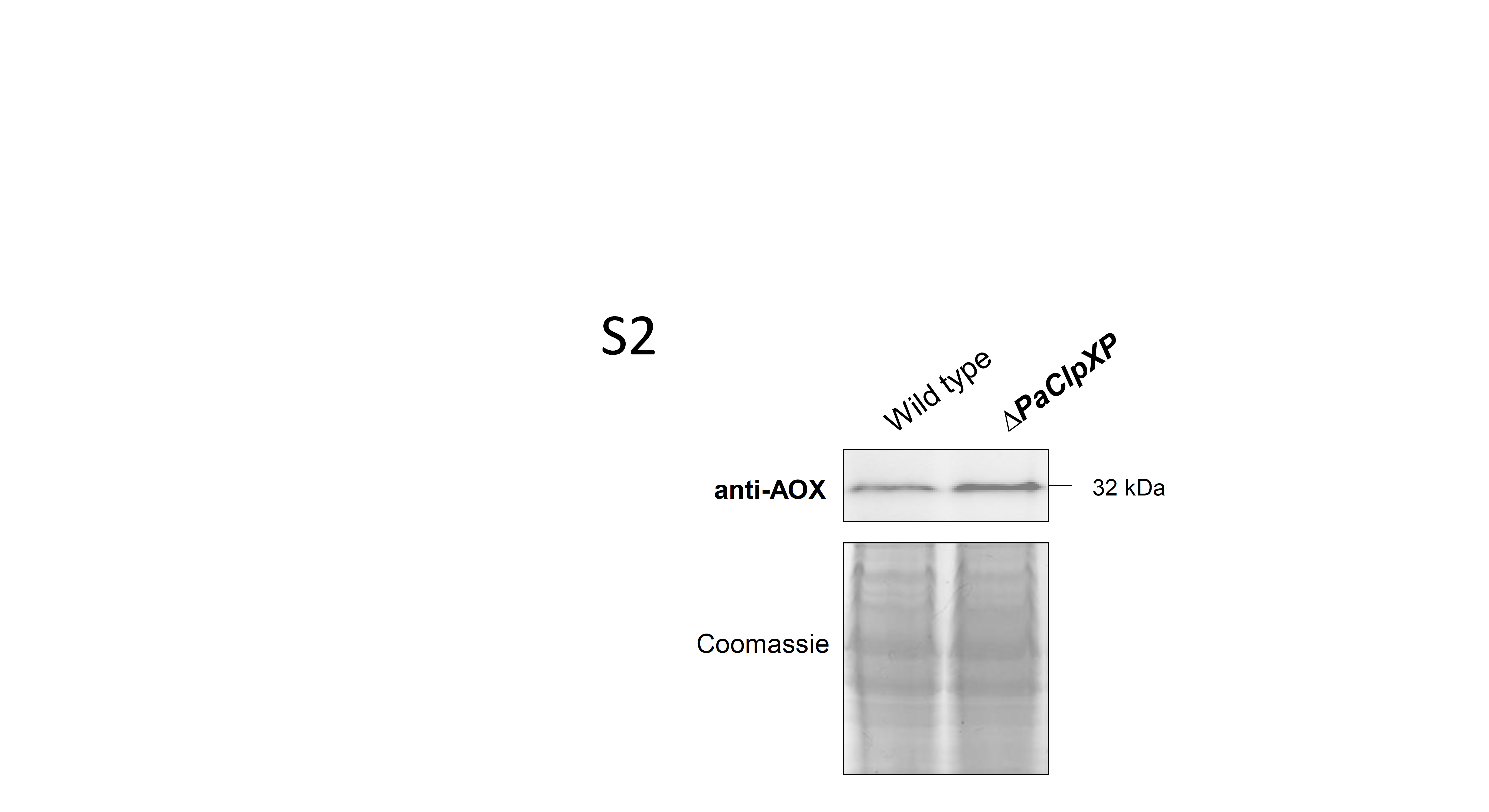


**Figure S2: Determination of the mitochondrial AOX protein amount in *∆PaClpXP* and the wild type.** Representative Western blot analysis of mitochondrial protein extracts from *∆PaClpXP* mutant strains (7d) and the *P. anserina* wild type (7d). Incubation with an anti-AOX (alternative oxidase) antibody showed an increased protein amount in the mutant compared to the wild type. The Coomassie stained gel serves as a loading control.

**Figure S3**


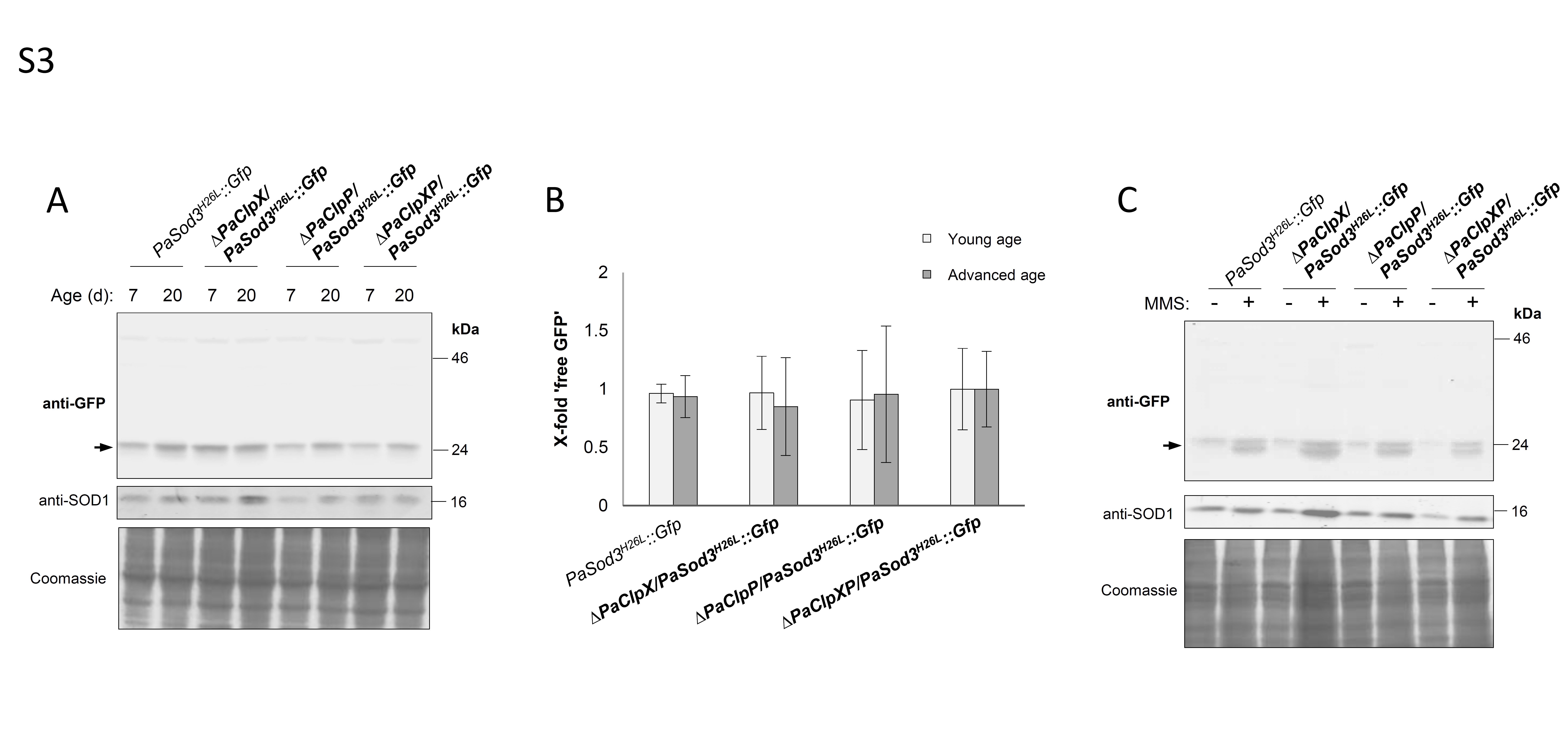


**Figure S3: Western blot analysis using the *PaSod3^H26L^::Gfp* mitophagy reporter strain.** (**A)** Monitoring mitophagy by western blot analysis of 7 and 20 days old *PaSod3^H26L^::Gfp* compared to ∆*PaClpX*/*PaSod3^H26L^::Gfp, ∆PaClpP/PaSod3^H26L^::Gfp* and *∆PaClpXP*/*PaSod3^H26L^::Gfp*. (**B**) Quantification of “free GFP” protein levels of 7 (n= 4) and 20 (n= 4) days old *PaSod3^H26L^::Gfp* vs. ∆*PaClpX*/*PaSod3^H26L^::Gfp, ∆PaClpP/PaSod3^H26L^::Gfp and ∆PaClpXP/PaSod3^H26L^::Gfp* normalized to the level of PaSOD1. Protein abundance in 7 days old *PaSod3^H26L^::Gfp* was set to 1. Error bars correspond to the standard deviation. *P* values were determined by student’s t test.  (**C**) Monitoring mitophagy during MMS treatment (0.09 % for the last 5 h of cultivation) by western blot analysis of 7 d old *PaSod3^H26L^::Gfp* compared to *∆PaClpX/PaSod3^H26L^::Gfp, ∆PaClpP/PaSod3^H26L^::Gfp* and *∆PaClpXP*/*PaSod3^H26L^::Gfp*.

**Figure S4**


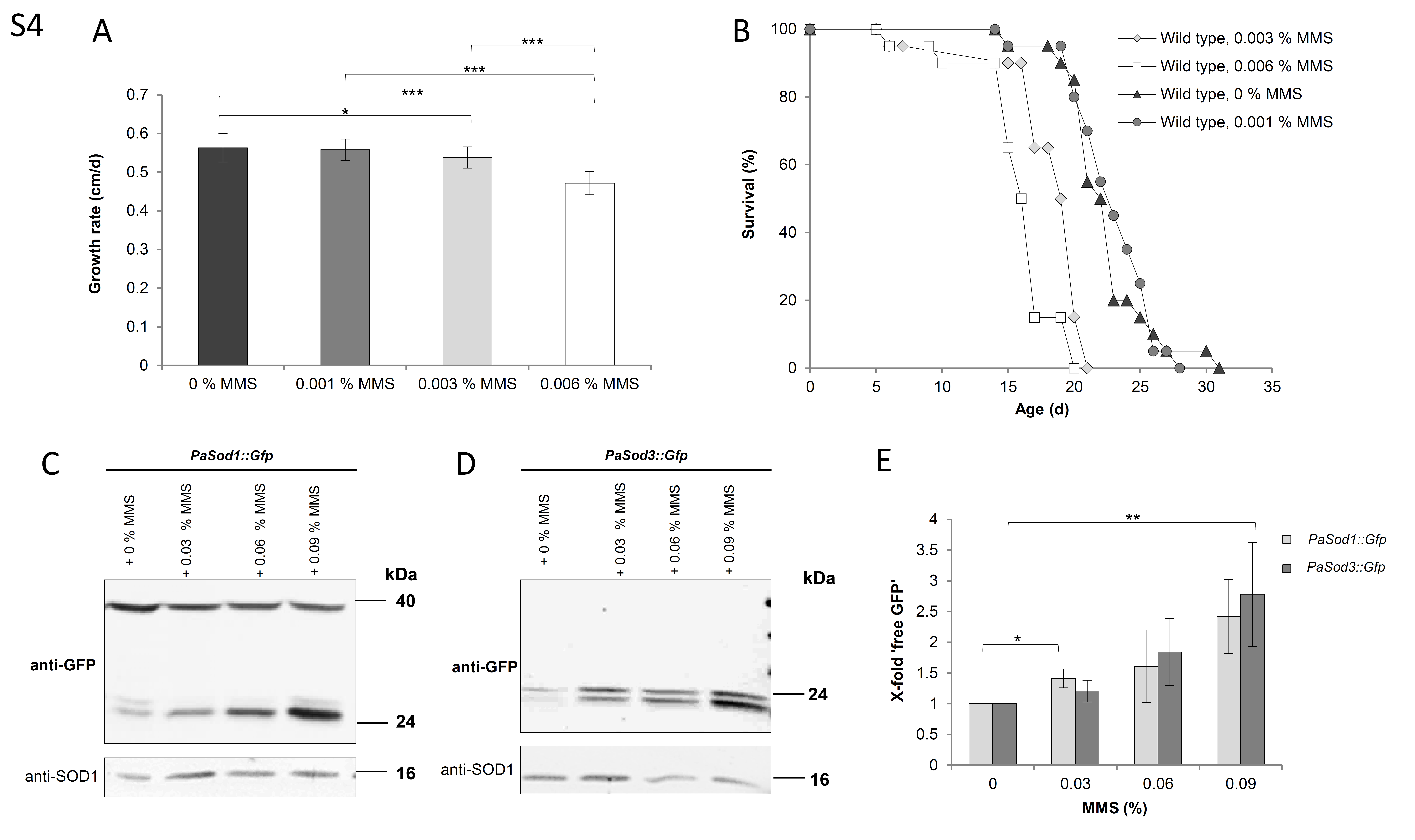


**Figure S4: Methyl methanesulfonate (MMS) as a tool to study mitophagy induction in *P. anserina*.** (**A**) Growth rates of the wild type (n= 19) cultivated on M2 agar medium under 0, 0.001, 0.003 and 0.006 % MMS treatment. *P*-values were determined by two-tailed Mann-Whitney-Wilcoxon U test. Error bars correspond to the standard deviation. (**B**) Survival curves of the wild type (n= 19) under 0, 0.001, 0.003 and 0.006 % MMS treatment. *P*-values were determined between 0 and 0.001 % MMS (*P* < 0.001), 0 and 0.003 % MMS (*P* < 0.001), 0 and 0.006 % MMS (*P* < 0.001) as well as between 0.001 and 0.003 % MMS (*P* < 0.001), 0.001 and 0.006 % MMS (*P* < 0.001) and between 0.003 and 0.006 % MMS (*P* > 0.05) by two-tailed Mann-Whitney U test. (**C**) Monitoring autophagy by western blot analysis of *PaSod1::Gfp* cultivated on CM liquid medium under 0, 0.03, 0.06 and 0.09 % MMS treatment. **(D**) Monitoring mitophagy by western blot analysis of *PaSod3::Gfp* cultivated on CM liquid medium under 0, 0.03, 0.06 and 0.09 % MMS treatment. (**E**) Quantification of ‘free GFP’ protein levels of *PaSod1::Gfp* (n= 4), respectively *PaSod3::Gfp* (n= 3) cultivated on CM liquid medium under 0, 0.03, 0.06 and 0.09 % MMS treatment normalized to the level of PaSOD1. Protein abundance in the untreated control strain was set to 1. Error bars correspond to the standard error. *P* values were determined by student’s t test.

**Figure S5**


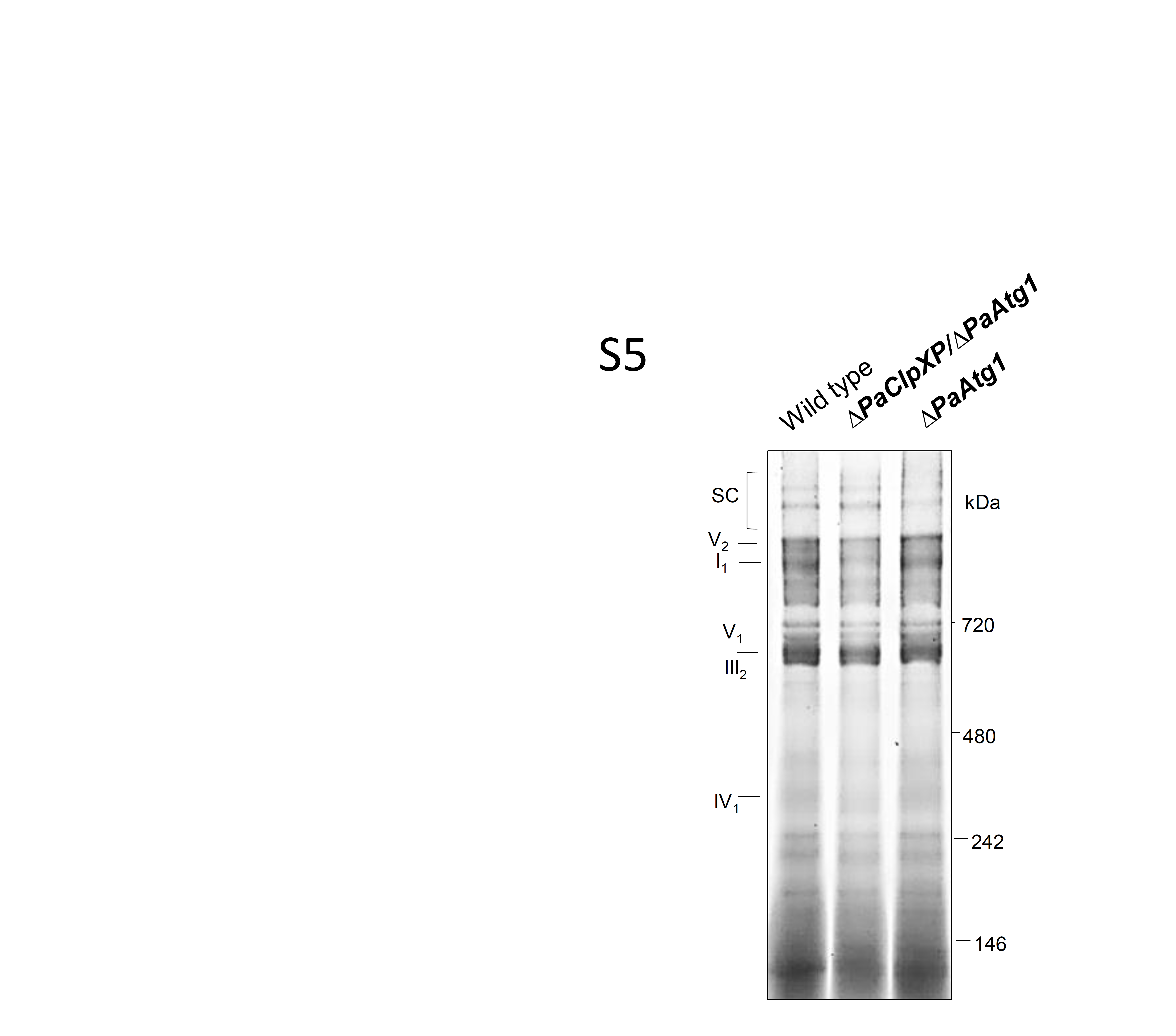


**Figure S5: BN-PAGE analysis of mitochondrial extracts from *∆PaClpXP/∆PaAtg1* and ∆*PaAtg1* compared to wild type*.*** Representative BN-PAGE analysis of 100 µg mitochondrial protein extracts from 7 days old wild type, *∆PaClpXP/∆PaAtg1* and ∆*PaAtg1* (3 mitochondrial preparations). SC= supercomplexes, V_2_= Complex V dimer; I_1_= Complex I monomer; V_1_= Complex V monomer; III_2_= Complex III dimer; IV_1_= Complex IV monomer.
